# Propensity to Obesity Impacts the Neuronal Response to Energy Imbalance

**DOI:** 10.3389/fnbeh.2015.00052

**Published:** 2015-02-26

**Authors:** Marc-Andre Cornier, Kristina L. McFadden, Elizabeth A. Thomas, Jamie L. Bechtell, Daniel H. Bessesen, Jason R. Tregellas

**Affiliations:** ^1^Division of Endocrinology, Metabolism and Diabetes, Department of Medicine, Anschutz Medical Campus, University of Colorado School of Medicine, Aurora, CO, USA; ^2^Anschutz Health and Wellness Center, University of Colorado Anschutz Medical Campus, Aurora, CO, USA; ^3^Department of Psychiatry, Anschutz Medical Campus, University of Colorado School of Medicine, Aurora, CO, USA; ^4^Department of Neuroscience, Anschutz Medical Campus, University of Colorado School of Medicine, Aurora, CO, USA; ^5^Research Service, VA Medical Center, Denver, CO, USA

**Keywords:** fMRI, neuroimaging, overweight, insula, prefrontal cortex, underfeeding, overfeeding

## Abstract

The mechanisms responsible for the propensity to gain weight or remain normal weight are poorly understood. The objective of this study was to study the neuronal response to visual food cues during short-term energy imbalance in healthy adults recruited as obesity-resistant (OR) or obesity-prone (OP) based on self-identification, body mass index, and personal/family weight history. Twenty-five OR and 28 OP subjects were studied in underfed (UF) and overfed (OF) as compared to eucaloric (EU) conditions in a randomized crossover design. Each study phase included a 3-day run-in diet, 1 day of controlled feeding (basal energy needs for EU, 40% above/below basal energy needs for OF/UF), and a test day. On the test day, fMRI was performed in the acute fed stated (30 min after a test meal) while subjects viewed images of foods of high hedonic value and neutral non-food objects. Measures of appetite and hormones were also performed before and every 30 min after the test meal. UF was associated with significantly increased activation of insula, somatosensory cortex, inferior and medial prefrontal cortex (PFC), parahippocampus, precuneus, cingulate, and visual cortex in OR. However, UF had no impact in OP. As a result, UF was associated with significantly greater activation, specifically in the insula, inferior PFC, and somatosensory cortex in OR as compared to OP. While OF was overall associated with reduced activation of inferior visual cortex, no group interaction was observed with OF. In summary, these findings suggest that individuals resistant to weight gain and obesity are more sensitive to short-term energy imbalance, particularly with UF, than those prone to weight gain. The inability to sense or adapt to changes in energy balance may represent an important mechanism contributing to excess energy intake and risk for obesity.

## Introduction

Despite the high prevalence of overweight and obesity, some individuals appear to be resistant to weight gain and obesity even though they live in the same obesogenic environment. An individual’s susceptibility to weight gain may be associated with a greater ability to adapt to changes in energy balance. We have previously shown that thin, “obesity-resistant” (OR) individuals are more sensitive to 2 days of overfeeding and to an acute meal than individuals prone to weight gain and obesity with greater changes in appetite-related behaviors and in neuronal responses to food cues (Cornier et al., 2004, 2009, 2013). How underfeeding, acute caloric reduction or acute overfeeding impacts the neuronal response to food cues, however, has not been well studied.

The regulation of energy balance is a result of complex interactions between physiologic signals, such as leptin and intestinal-derived peptides, and non-physiologic signals, such as reward, motivation, attention, and environmental cues (Zheng et al., 2009). Neuroimaging studies have helped elucidate neuroanatomical and neurophysiologic correlates associated with food intake regulation and how these processes may be altered in obesity. Neuroimaging studies have focused on how obesity impacts the brain’s response to food-related cues and have generally found altered responses to visual, gustatory, and olfactory cues in brain regions important in the regulation of appetitive regulation (Rothemund et al., 2007; Rosenbaum et al., 2008; Stoeckel et al., 2008; Cornier et al., 2009; McCaffery et al., 2009; Martin et al., 2010; Carnell et al., 2012; Pursey et al., 2014). It is unclear, though, if these findings are a consequence or cause of obesity (Rothemund et al., 2007; Stoeckel et al., 2008; Martin et al., 2010). We have shown that both reduced-obese (obese individuals who were studied after 8–10% weight loss through caloric restriction) and obesity-prone (OP) individuals not only have altered eating related behaviors but also altered neuronal responses to visual food cues in response to feeding as compared to thin, OR individuals (Cornier et al., 2004, 2009, 2013; Thomas et al., 2013). These studies suggest that risk for weight gain and obesity is not only associated with changes in the neuronal response to food but also in an inability to alter this response based on physiologic need.

Studies examining the neuronal response to short-term changes in energy balance have not been well examined, especially in individuals resistant or prone to weight gain and/or obesity. We therefore designed the present study to assess the neuronal response to visual food cues during short-term energy imbalance, 1-day of under- and over-feeding, in thin individuals who identified themselves as being OR, as compared to “never-obese” OP individuals. Research participant were classified as OR or OP based on personal and family weight history as well as the subject’s own perception of their tendency to gain weight or not, as previously defined (Schmidt et al., 2012, 2013; Smucny et al., 2012; Cornier et al., 2013; Thomas et al., 2013, 2014). We hypothesized that OR individuals would be more sensitive to changes in short-term energy balance with greater changes in the neuronal response to food cues with under- and overfeeding vs. eucaloric (EU) feeding as compared to OP individuals.

## Materials and Methods

### Research participants

This study was conducted according to the principles of the Declaration of Helsinki and was approved by the Colorado Multiple Institutional Review Board. All research participants provided written informed consent.

As we have previously described, subjects were recruited to have a propensity to be resistant to weight gain and obesity (OR) or to be prone to weight gain and obesity (OP) (Schmidt et al., 2012, 2013; Smucny et al., 2012; Cornier et al., 2013; Thomas et al., 2013, 2014). Subjects were 25–40 years of age and were free of significant medical and psychiatric disease, including eating disorders as assessed by screening medical history, physical examination, biochemical testing, and questionnaires [eating attitudes test (Garner et al., 1982) and the Center for Epidemiologic Studies Depression Scale (Radloff, 1977)]. OR subjects responded to advertisements for “naturally thin people.” They defined themselves as “constitutionally thin” based on their perception of difficulty gaining weight, expending little effort to maintain their weight, and reporting a sense that their body weight regulation was somewhat “different” from other people. They had a body mass index (BMI) of 17–25 kg/m^2^ and reported no obese first degree relatives, never being overweight themselves, weight stability despite few to no attempts to lose weight, and no high levels of physical activity. OP subjects, in contrast, responded to advertisements for “people who struggle with their weight” and defined themselves as chronically struggling with body weight control. They had a BMI of 20–30 kg/m^2^and reported at least one obese first degree relative, a history of weight fluctuations despite putting effort into not gaining weight and previous attempts to lose weight, but were not actively attempting to lose weight and were weight stable for at least 3 months. All participants were right-handed and could not have contraindications for MRI scanning. A total of 28 subjects were studied in each group. Data from three individuals were not included in the analysis due to technical problems or head movement of >2 mm during scanning data. As a result, 25 OR (14 men, 11 women) and 28 OP individuals (14 men, 14 women) are included in the current analyses.

### Study design and measurements

**Figure 1:**
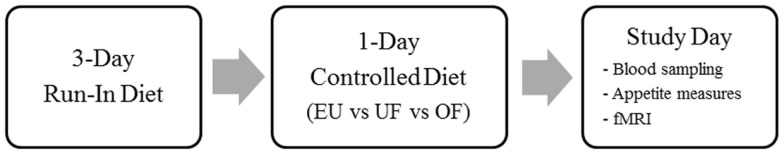
**Study design**. Subjects were studied during three conditions, eucaloric (EU), underfeeding (UF), and overfeeding (OF) as shown.

As previously described (Cornier et al., 2013; Thomas et al., 2013, 2014), subjects first underwent baseline assessments, including anthropometric measurements (body weight, height), the three factor eating questionnaire (Stunkard and Messick, 1985), and body composition (lean body mass, fat mass) measurement by dual-energy x-ray absorptiometry (DPX whole-body scanner, Lunar Radiation Corp., Madison, WI, USA). Each subject then underwent three study phases in a randomized counterbalanced manner, with each phase consisting of a 3-day baseline EU diet period to ensure energy and macronutrient balance, followed by the intervention diet on day 4, and then a study day on day 5 as shown in Figure 1. The three study phases consisted of one of the following intervention diets on day 4: EU diet, overfeeding (OF) by 40% above estimated energy needs, or underfeeding (UF) by 40% of baseline caloric intake. During all three study periods, the diets were made up of the same macronutrient composition (50% carbohydrate, 30% fat, and 20% protein). Estimates of daily energy needs were made using lean body mass (as determined by DEXA) in the following equation: resting metabolic rate = (fat free mass × 23.9) + 372. The estimates were confirmed using resting metabolic rate as assessed by indirect calorimetry and multiplied by an activity factor of 1.3. This method has been used successfully by our group in a number of prior studies (Cornier et al., 2004, 2006, 2007, 2009; Adochio et al., 2009; Wang et al., 2012). All food was prepared and provided by the Clinical Translational Research Center metabolic kitchen. Subjects presented to the Clinical Translational Research Center each morning, were weighed, ate breakfast, and picked up the remainder of their daily meals, which were packed in coolers. Subjects were asked to maintain their usual pattern of physical activity and were regularly questioned regarding activity and compliance. Subjects were asked to not consume any alcoholic or calorie-containing beverages during the study period. In women, study days were scheduled during the follicular phase of their menstrual cycle.

### Study day

Again as previously described (Cornier et al., 2013; Thomas et al., 2013, 2014), subjects presented to the outpatient clinic of the Clinical Translational Research Center after an overnight fast of at least 10 h. They first completed baseline (fasting) appetite ratings by visual analog scale (VAS) (Cornier et al., 2004). Hunger was rated by VAS on a line preceded by the question, “How hungry are you right now?” and anchored on the left by “not at all hungry” and by “extremely hungry” on the right. Satiety was rated by the question, “How full do you feel right now?” with the anchors “not at all” and “extremely.” An intravenous catheter was then placed for blood sampling. Baseline (fasting) samples were drawn for hormones [insulin, leptin, ghrelin, peptide YY (PYY), glucagon like peptide-1 (GLP-1)] and metabolites (glucose, triglycerides, free fatty acids). Subjects were then escorted to the Brain Imaging Center at the University of Colorado where they consumed a liquid breakfast meal over 20 min. The caloric content of the liquid breakfast was equal to 25% of the energy provided during the intervention diet (EU, OF, or UF) and had an identical macronutrient composition. fMRI measures (described below) were then performed 60 min after the start of the test meal. Repeat appetite ratings by VAS and blood sampling were also performed 30, 60, 90, 120, 150, and 180 min after the meal.

### Laboratory analyses

Blood samples were collected in EDTA-containing tubes, centrifuged, placed in aliquot tubes and stored at −70 to −80°C until analysis. All assays were run after all three studies phases were complete for each subject. For GLP-1, 30 μl of dipeptidyl peptidase IV inhibitor was added to the 4 ml EDTA tube prior to collection. Total GLP-1 assays were performed with Alpco Diagnostics ELISA (43-GPTHU-E01). Insulin concentrations were measured using competitive radioimmunoassay (Millipore). Radioimmunoassays were used to analyze serum leptin (Millipore), serum PYY concentrations (Millipore Cat. #PYYT-66HK), and total serum ghrelin concentrations (Millipore Cat. #GHRT-89HK). All radioimmunoassays were performed with a Perkin Elmer Wallac Gamma counter using Maciel RIA-AID data reduction software. Assays for glucose, triglycerides, and free fatty acids were performed on the Olympus AU400e Chemistry Analyzer (Beckman).

### Functional magnetic resonance imaging

As previously described (Cornier et al., 2013), imaging studies were performed using a General Electric (Milwaukee, WI, USA) 3.0 T MR scanner with a standard quadrature head coil. Prior to functional imaging, high-resolution, T1-weighted 3D anatomical scan over 10 min was acquired for each subject. Functional images were then acquired with an echo-planar gradient-echo T2* blood oxygenation level dependent (BOLD) imaging contrast technique, with TR = 2000 ms, TE = 30 ms, 64^2^ matrix, 240 mm^2^ FOV, 27 axial slices angled parallel to the planum sphenoidale, 2.6 mm thick, 1.4 mm gap. Additionally, one inversion-recovery echo-planar-image (TI = 505 ms) volume was acquired to improve coregistration between the echo-planar images and gray matter templates used in pre-processing. The acquisition voxel size was 3.43 mm × 3.43 mm × 2.6 mm. Head motion was minimized with a VacFix head-conforming vacuum cushion (Par Scientific A/S, Odense, Denmark). Functional imaging was performed while the participants were presented visual stimuli using a projector and screen system. Previously validated visual stimuli consisted of three different categories: neutral non-food-related objects, foods of high hedonic value, and foods of neutral hedonic value (Burger et al., 2011). To reduce the potential for habituation, different but similar images were used in each of the scanning sessions (EU, OF, UF). Because previous studies have shown that comparisons involving neutral food objects to be qualitatively similar but less sensitive (Cornier et al., 2007), the primary analysis examined differences between hedonic foods and non-food objects. Images of hedonic foods included images of pastries, savory dishes, furti, and images of neutral foods included images of bread products, vegetables, and starchy side dishes. Images of non-food objects included images of sceneries, furniture, buildings, tools, vehicles, books, and others. Two runs were performed with each run consisting of a pseudo-randomized block design with six blocks of pictures of each category. Seven blocks of a low-level baseline (fixation cross) were also included in each run. Each block consisted of four stimuli shown for 4 s each for a total of 16 s/block. Four additional scans were acquired at the beginning of each run to minimize saturation effects. Subjects were asked lie quietly and to view the images.

### Calculations and statistical analyses

fMRI data were analyzed using Statistical Parametric Mapping 8 software (SPM8, Wellcome Department of Imaging Neuroscience, London, England) as previously described (Cornier et al., 2013). Data analyses were blind to participant group. Data from each subject were realigned to the first echo-planar image, normalized to the Montreal Neurological Institute (MNI) template, using a gray-matter-segmented IR-EPI as an intermediate to improve registration, and smoothed with a 6 mm FWHM Gaussian kernel. Movement parameters derived from the realignment procedure were included in the model to reduce the effects of residual motion-related noise. The hemodynamic response was modeled with a double gamma function, without temporal derivatives, using the general linear model in SPM8. A 128 s high pass filter was applied to remove low-frequency fluctuation in the BOLD signal. To account for both within-group and within-subject variance, a random effects analysis was implemented. Parameter estimates for each individual’s first level analysis (SPM contrast images) contrasting “hedonic food cues” to “non-food objects” were entered into second-level repeated measures ANOVA. Comparisons across conditions (e.g., underfed-EU) and group (OR–OP) were evaluated with directional contrasts (SPM *t*-contrasts). Results were considered significant at a whole-brain level if they exceeded a voxel-wise threshold of *p* < 0.01 and a cluster-level false discovery rate (FDR) threshold of *q* < 0.05 (critical cluster size = 193). Results in all figures are statistical parametric maps (i.e., colored voxels indicate *t*-values), thresholded at the above level, overlaid on a group averaged anatomical image. Finally, order effect was examined using SPSS v21 (IBM Corp, Armonk, NY).

Non-imaging analyses were performed using SigmaStat software (Jandel Scientific, San Rafael, CA, USA). The total area under the curve (AUC) for appetite ratings and biochemical measurements was calculated using the Trapezoid Method (Allison et al., 1995), using all time points over 3 h post-test meal. Group differences were analyzed using a two-sided *t*-test with significance set at a level of 0.05. Finally, the Pearson Product Correlation between the fMRI BOLD% signal change, relative to the global mean (local maxima) and appetite/biochemical measures was examined, with a significance set at a level of 0.05, Bonforoni corrected for the number of brain regions examined.

## Results

### Subject characteristics

Twenty-five OR and 28 OP subjects were studied (Table 1). Compared to OR subjects, OP subjects had higher BMI, body fat mass, and percent body fat but had similar fat free mass. As previously described, OP subjects had higher scores for restraint, disinhibition, and hunger on the Three Factor Eating Questionnaire than OR, but no significant group differences in ratings of hunger or satiety at baseline (EU) or in response to energy imbalance (OF, UF) were observed (Thomas et al., 2013). Also as previously reported, OR had lower leptin (OR: 598 ± 71 ng/ml, OP: 1,881 ± 72 ng/ml, *p* < 0.001) and insulin (OR: 6,908 ± 452 ng/ml, OP: 9,147 ± 436 ng/ml, *p* < 0.05) AUC and higher ghrelin (OR: 147,256 ± 2,286 ng/ml, OP: 124,586 ± 2,207 ng/ml, *p* < 0.05) AUC than OP at baseline, but other hormones (PYY, GLP-1) and metabolites (glucose, free fatty acids, triglycerides) were similar between groups (Thomas et al., 2014).

**Table 1:** **Baseline characteristics**.

	OR	OP
Total *n* (male/female)	25 (14/11)	28 (14/14)
Age (years)	30.7 ± 3.4	30.4 ± 3.9
BMI (kg/m^2^)	20.9 ± 1.9	26.1 ± 2.8^a^
Lean body mass (kg)	48.5 ± 10.3	53.4 ± 10.4
Fat mass (kg)	10.7 ± 3.6	22.7 ± 8.0^a^
Percent body fat	18.8 ± 4.6	28.7 ± 8.0^a^

*Mean ±SD for obese resistant (OR) and obese prone (OP)*.

*^a^*p* < 0.001*.

### Effects of underfeeding

**Figure 2:**
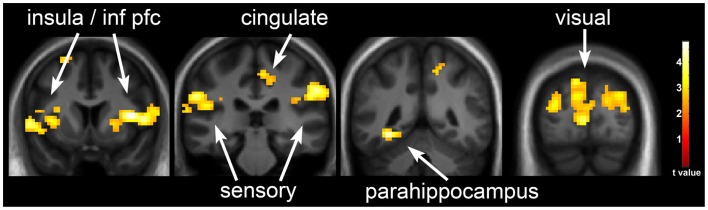
**Neuronal response to visual food cues in obesity-resistant (OR) individuals with underfeeding (UF)**. The neuronal response to visual stimuli of foods of high hedonic value as compared to non-food objects with UF as compared to EU in OR is shown. Robust activation is observed in the insula, inferior prefrontal cortex, medial prefrontal cortex, somatosensory cortex, cingulate cortex, parahippocampus, and visual cortex. Statistical maps thresholded at a voxel-wise threshold of *p* < 0.01 and a cluster-level false discovery rate (FDR) threshold of *q* < 0.05 and overlaid onto the group averaged anatomical image. Data are shown in the neurological convention (right hemisphere on the right).

**Figure 3:**
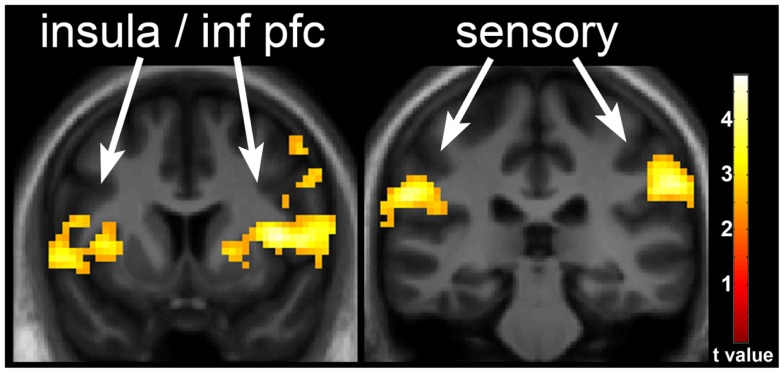
**Neuronal response with underfeeding (UF) in obesity-resistant (OR) as compared to obesity-prone (OP) individuals**. The difference in neuronal response with UF as compared to EU in OR as compared to OP individuals when viewing foods of high hedonic value is shown. Greater response is seen in the insula, inferior prefrontal cortex, and somatosensory cortex in OR as compared to OP individuals. Statistical maps thresholded at a voxel-wise threshold of *p* < 0.01 and a cluster-level false discovery rate (FDR) threshold of *q* < 0.05 and overlaid onto the group averaged anatomical image. Data are shown in the neurological convention (right hemisphere on the right).

**Figure 4:**
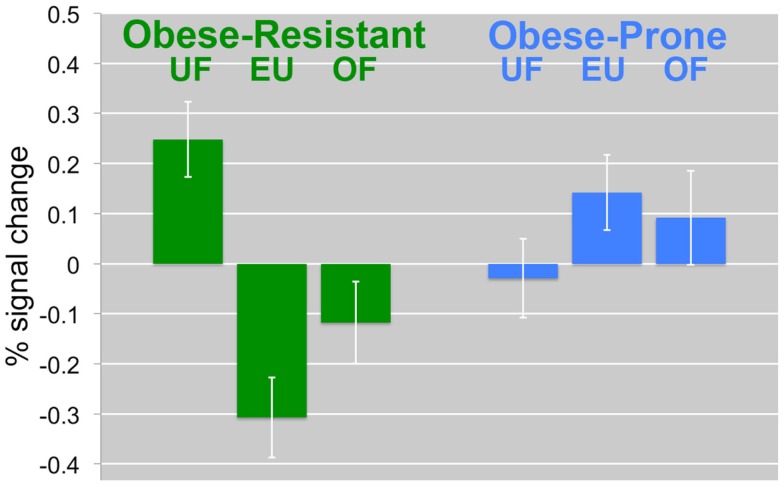
**Change in the insula/inferior prefrontal cortex by diet and group**. The percent signal change in the insula/inferior prefrontal cortex is compared between diet intervention (UF, EU, OF) and group (OR, OP) is shown. In OR, UF was associated with a significant increases in response. In OP, the opposite effects were observed. Mean BOLD responses (±SEM) are shown for the insula/inferior prefrontal cortex.

One day of underfeeding (UF; 40% below basal needs) as compared to EU feeding resulted in increased neuronal response to visual food cues in bilateral insula, somatosensory cortex, inferior prefrontal cortex (PFC), and visual cortex as well as left medial PFC, parahippocampus and precuneus and right cingulate in OR (Table 2; Figure 2). UF, however, had no impact on the neuronal response to food cues in OP. As a result, directional *t*-contrasts revealed significantly greater neuronal response in OR as compared to OP (UF > EU, OR > OP) in insula, inferior PFC, and somatosensory cortex (Table 2; Figure 3). The signal change in the insula/inferior PFC is shown in Figure 4. Order effect was examined for the change in insula/inferior PFC and none was found (*p* = 0.63). To assess whether these differences were due to differences in the attention network we examined whether there were group differences in response to the non-food images and found no differences.

**Table 2:** **Coordinates and brain regions showing differential responses to diet conditions between OR and OP**.

Brain region	MNI coordinates^a^			*T* value^b^	Cluster size
	*x*	*y*	*z*	
**OR, underfed > eucaloric**
Insula/inferior prefrontal cortex (R)	39	11	10	4.78	982
Somatosensory cortex (R)	60	−28	34	4.26	
Inferior prefrontal cortex (R)	54	5	4	4.31	
Inferior prefronal cortex (L)	−57	5	1	4.67	745
Insula (L)	−36	5	4	3.77	
Medial prefrontal cortex (L)	−12	−4	61	4.01	
Parahippocampus (L)	−33	−46	−8	4.42	635
Precuneus (L)	−12	−88	40	4.15	
Visual cortex (L)	−3	−85	4	3.91	
Visual cortex (R)	33	−79	16	3.27	216
Visual cortex (R)	24	−79	16	3	
Somatosensory cortex (L)	−45	−28	19	4.17	231
Somatosensory cortex (L)	−54	−34	−28	4.17	
Cingulate cortex (R)	12	−25	40	3.36	193
Cingulate cortex (R)	6	−31	49	3.17	
**OR > OP, underfed vs. eucaloric interaction**
Insula/inferior prefrontal cortex (R)	42	11	7	4.55	1314
Inferior prefronal cortex (R)	57	14	7	4.15	
Inferior prefronal cortex (L)	−54	2	7	4.25	531
Somatosensory cortex (R)	60	−28	31	4.16	201
Somatosensory cortex (L)	−57	−34	28	4.07	233

*All values in table are significant at a voxel-wise threshold of *p* < 0.01 and a cluster-corrected FDR threshold of *q* < 0.05*.

*^a^Stereotactic coordinates in MNI space*.

*^b^T values reported for local maxima within clusters*.

### Effects of overfeeding

One day of overfeeding (OF; 40% above basal needs) as compared to EU feeding resulted in decreased neuronal response to visual food cues in posterior temporal visual cortex in both OR and OP when grouped together (right, *x* = 48, *y* = −76, *z* = −2, *t* = 5.2, *p* = 0.001; left, *x* = −51, *y* = −70, *z* = −8, *t* = 4.04, *p* = 0.038). No group (OR vs. OP) differences were observed, however, in the response to OF.

### Correlates of neuronal response

Because there were differences in BMI between groups as per group definitions, it was determined that these variables should not be included in the analyses when OR/OP status was included. However, we did repeat the analyses adjusting for fat mass. These models indicated that fat mass did not have a significant effect on any of the outcomes. We did not find a correlation between neuronal responses to food cues and appetite ratings or measures of eating behaviors such as restraint and disinhibition. We did find, however, that changes in PYY in response to UF were positively correlated to changes in inferior PFC/insula activation (*r* = 0.53, *p* = 0.005).

## Discussion

This study was completed to investigate the neuronal response to visual food cues in response to short-term energy imbalance (1 day of under- and over-feeding) in healthy adult subjects screened to be resistant to weight gain and obesity (OR) as compared to individuals screened to be prone to weight gain and obesity (OP). Underfeeding was associated with increased neuronal response to food cues in OR as compared to OP. Overfeeding, however, was not associated with group (OR/OP) differences. These results suggest that individuals resistant to weight gain are more sensitive to short-term changes in energy balance especially with underfeeding than individuals prone to weight gain and obesity. These results were not influenced by fat mass, suggesting that the differences seen may represent an important mechanism for the propensity to weight gain and obesity and are not simply a result of increased body fat.

We have previously shown that, while thin or normal weight individuals show greater activation of visual cortex, overall the neuronal response to food cues during the overnight fasted state is similar between individuals resistant or prone to obesity (Cornier et al., 2009). Two days of overfeeding, however, is associated with significant attenuation of a network of brain regions, including the insula, in thin as compared to reduce-obese individuals (Cornier et al., 2009). Furthermore, a single meal also significantly attenuates the response to food cues in the insula and PFC in OR as compared to OP individuals (Cornier et al., 2013). Recent meta-analyses have also found that obese individuals appear to have great activation in the PFC, insula, and caudate when fed as compared to normal weight individuals (Kennedy and Dimitropoulos, 2014; Pursey et al., 2014). These findings suggest that individuals who are prone to obesity and who already are obese do not seem to be as sensitive to food cues during times of acute and short-term positive energy balance. This could also represent a mechanism for the consistent under-reporting of food intake in obese individuals.

Fasting studies have shown greater activation of reward centers in normal weight individuals (Goldstone et al., 2009; Siep et al., 2009). Chronic caloric restriction in obese individuals, resulting in weight loss, has also been shown to increase activation in reward and regulatory brain centers (Rosenbaum et al., 2008) but this is in the setting of significantly reduced body weight, which may have independent effects. We are not aware, however, of any published studies that have examined the effects of acute caloric restriction or underfeeding on the neuronal response to food cues. The current findings suggest that OR individuals appear to be more sensitive to short-term negative energy balance than OP. This may be a result of their reduced fuel stores, although adjusting for fat mass did not impact the results. The specific mechanisms for these findings of differences in neuronal “sensitivity” to energy imbalance are not entirely clear. Certainly, observed effects could be driven by baseline genetic predispositions. Learned behaviors could also be important. Finally, while adjusting for fat mass did not impact the results, it is still possible that years of slight overnutrition resulting in higher body and fat mass may have led to changes in the brain response to food-related cues in the OP. Moreover, there are certainly data to support that overnutrition may lead to desensitization of the system (Cornier et al., 2009, 2013), and as discussed below alterations in insula activity may play an important role.

We have previously shown that 2 days of overfeeding was associated in significant differences in the neuronal response to similar visual food cues in reduced-obese as compared to thin individuals (Cornier et al., 2009). In the present study, however, we did not find a difference between OR and OP after 1 day of overfeeding. This lack of a difference in response to overfeeding is likely due to the fact that in the previous studies participants were studied in the fasted state while in the present study they were studied in the acute fed state. As seen in Figure 4, neuronal activation is significantly reduced even in the EU state in the OR suggesting significant sensitivity to even the acute positive energy balance associated with a meal. The OP has an increased activation after both EU and overfed meals. Furthermore, the meal effect may have been too great to see an effect from this modest 40% overfeeding.

We and others have found that food cues have a robust impact on the insula (Gordon et al., 2000; Hinton et al., 2004; Pelchat et al., 2004; Cornier et al., 2007, 2009, 2013; Van Der Laan et al., 2011; Tang et al., 2012; Pursey et al., 2014). Again, we show the effects of phenotype and energy balance on the response to visual food cues appear to be strongest in these brain regions. The insula has many known functions that relate to eating behaviors, including being important for the memory of the rewarding aspects of food (Levy et al., 1999). The insula may also be a key center for the interpretation of bodily states and peripheral signals (Augustine, 1996), and the greater activation seen in OR with underfeeding may thus relate to a greater sensitivity to changes in homeostasis. One such peripheral signal could be the response to the hormone PYY. We saw a correlation between changes in PYY and changes in activation in the insula with underfeeding, and PYY has been shown to modulate insula activity (De silva et al., 2011).

An important strength of this study is that it was carried out individuals who were not yet obese but were selected for a propensity to gain weight or to remain thin; however, classifying individuals as being prone or resistant to obesity before its development is associated with problems. We cannot be certain, though, that OP individuals may consciously alter their behaviors to reduce weight gain, nor can we be certain that OR individuals may experience health-related problems or life events that may result in weight gain. We have previously shown, however, that these groups as identified are associated with other meaningful biologic and behavioral differences (Schmidt et al., 2012, 2013; Smucny et al., 2012; Cornier et al., 2013; Thomas et al., 2013, 2014). We are currently collecting longitudinal weight data, which will ultimately determine whether or not these categories are valid. We will also evaluate individuals for behaviors and/or events that may impact longitudinal weight. Furthermore, because the OP individuals had higher BMI and fat mass than the OR individuals, it is possible that the group differences seen may be related to the greater fat mass and not the classification of obesity proneness, *per se*. Adjusting the data for fat mass, however, did not alter the findings; as such, we would argue that these effects are not likely to be due to higher adiposity and therefore are a marker of potential obesity risk. Finally, our conservative whole-brain analysis approach, resulting in a cluster extent threshold of 193 voxels, may have decreased the sensitivity to see differences in other regions found to be involved by others, such as the amygdala, orbital frontal cortex, striatum, and nucleus accumbens (Tang et al., 2012).

In conclusion, the results of this study suggest that individuals who are prone to weight gain have differences in brain regions known to be important in body weight regulation as compared to those who are recruited to be resistant to weight gain and obesity. Specifically, OR individuals had significant increases in neuronal responses to visual food cues in response to 1 day of underfeeding as compared to OP individuals. Paired with our previous findings that an acute meal attenuates the neuronal response to food cues in OR as compared to OP, these findings suggest that OR individuals are more sensitive to acute/short-term changes energy balance. These differences in the neuronal response to food cues between individuals of differing propensity for weight gain may represent a core feature of weight gain and obesity risk and an ability for those resistant to weight gain to adapt to changes in energy balance.

## Conflict of Interest Statement

The authors declare that the research was conducted in the absence of any commercial or financial relationships that could be construed as a potential conflict of interest.
